# Dose-Escalated Intensity-Modulated Radiotherapy for the Management of Locally Advanced Cervical Cancer

**DOI:** 10.7759/cureus.25965

**Published:** 2022-06-15

**Authors:** Balaji Shewalkar, Asfiya Khan, Dnyanda Yerlekar, Jitendra Patel, Hrishikesh Khadilkar, Rajakumar Sakthivel, Reeta Kataruka

**Affiliations:** 1 Radiation Oncology, Government Medical College and Cancer Hospital, Aurangabad, IND; 2 Preventive Oncology, Government Medical College and Cancer Hospital, Aurangabad, IND; 3 Pathology, Government Medical College and Cancer Hospital, Aurangabad, IND

**Keywords:** carcinoma cervix, brachytherapy, imrt, radiotherapy, dose escalation

## Abstract

Objective

In this study, we aimed to assess the response and toxicity related to dose escalation in external beam radiation therapy (EBRT) using intensity-modulated radiation therapy (IMRT) with weekly concurrent cisplatin followed by de-escalated brachytherapy (BT) in locally advanced carcinoma cervix of International Federation of Gynecology and Obstetrics (FIGO) 2018 stage IIB-IIIC1.

Materials and methods

Fifty-two patients diagnosed with cervical cancer FIGO 2018 stage IIB-IIIC1 were treated with curative intent from November 2019 to October 2021. The dose of 50 Gy was prescribed for the primary tumor volume and elective pelvic nodal volume followed by a primary boost to a dose of 20 Gy. Gross lymph node (LN) of size >1 cm after EBRT completion received a sequential nodal boost of 10 Gy. All patients received concurrent cisplatin to a dose of 40 mg/m^2^ for a total of five to six weekly cycles. All patients received two fractions of BT to a dose of 6 Gy after EBRT completion. Radiation-induced acute toxicities were graded according to the Radiation Therapy Oncology Group (RTOG) criteria and hematologic toxicity was graded according to the Common Terminology Criteria for Adverse Events (CTCAE v4.0).

Results

A median follow-up of six months was available for the 40 eligible patients. All patients tolerated treatment with an acceptable toxicity profile. Grade III dermatitis, grade III gastrointestinal (GI) toxicity, and grade III genitourinary (GU) toxicity were seen in three (7.5%), six (17.5%), and three patients (7.5%) respectively. Grade I anemia was evident in all patients. At six months after EBRT completion, 37 patients (92.5%) had a complete response and only three patients (7.5%) had residual disease.

Conclusion

Based on our findings, patients with cervical cancer treated with dose-escalated IMRT have a satisfactory outcome with reasonably low levels of treatment-related acute GI and GU toxicities. The findings of the present study endorse the notion that the application of a high dose of external radiation to the pelvis by IMRT technique with image-guided delivery could be an acceptable alternative to the standard-dose management schedule.

## Introduction

Cervical cancer is a global public health concern among women, with an estimated 604,127 new cases and 3,41,831 deaths reported in 2020 [1]. Globally, it is the fourth leading cause of cancer-related deaths in women but ranks second in India despite now being recognizable in its early stages, with 77,348 annual deaths reported due to a lack of implementation of population-based cancer screening programs and human papillomavirus (HPV) vaccination [2].

A pelvic external beam radiation therapy (EBRT) dose of 50 Gy is more advantageous than that of 45 Gy in cervical cancers because it covers the whole low-risk clinical target volume (CTV) and some parts of high-risk CTV that may not be adequately covered through brachytherapy (BT) [3]. BT in cervical carcinoma safely delivers 80-85 Gy of biologically equivalent dose in 2-Gy fractions (EQD2) to the tumor periphery, whereas the central cervix receives even higher doses (>120-Gy EQD2) with rapid dose fall-off, thereby minimizing the dose to the adjacent organs at risk (OAR), namely the bladder, bowel, and rectum. This results in favorable local control rates of 100%, 96%, and 86% for IB, IIB, and IIIB stages of cervical cancer, respectively [4].

Three-dimensional conformal radiation therapy (3DCRT) is more beneficial than conventional therapy because it shapes the radiation beam according to the primary target, ensuring a more precise target coverage while maintaining OAR constraints [5,6]. With technological advancement, intensity-modulated radiation therapy with image-guided treatment delivery (IG-IMRT) has been commonly employed due to its low acute toxicity profile, that is, acute grade II gastrointestinal (GI) toxicity of 60% with intensity-modulated radiation therapy (IMRT) versus 91% with 3DCRT [7]. The National Comprehensive Cancer Network (NCCN) guidelines 2022 recommend the IMRT technique for EBRT delivery. A parametrial boost of 5-10 Gy can be considered in select cases with bulky residual parametrial or lateral pelvic wall disease after the completion of pelvic EBRT. Pelvic and para-aortic lymph node (LN) involvement is the most significant negative prognostic factor for locoregional recurrence and overall survival (OS). The acceptable strategies for a nodal boost in clinical practice are simultaneous integrated boost (SIB) and sequential boost (SEB). A target dose of 54-63 Gy is essential to effectively sterilize lymph nodal diseases [8,9].

Treatment-related toxicity has been observed in 84% of cervical cancer patients receiving CTRT when radiation therapy (RT) is planned with the conventional technique [10,11]. The intensity and severity of these adverse RT effects depend on patient characteristics, such as general build, comorbidities, treatment compliance, and treatment-related characteristics such as RT technique and the number of concurrent weekly cisplatin cycles administered [12].

BT applicator placement may be challenging due to multifactorial reasons such as distortion of the cervix anatomy after CTRT, insufficient primary tumor regression, lack of USG-guided intrauterine tandem placement, and a high cost of treatment in comparison with EBRT primary boost in low- and middle-income countries. Although BT treatment with the remote after-loading technique is an efficacious intervention, the chances of applicator sag still exist, leading to dosimetric discrepancies between the calculated dose and actual treatment delivery dose. This raises a concern regarding the actual dose delivered to point A and adjacent vital organs. The ABS guidelines 2012 recommended a dose to maximally exposed 2 cm^3^ of the organs at risk (OARsD2cc) dosimetric thresholds of 90 Gy for the urinary bladder and 75 Gy for the rectum and sigmoid colon [13]. Romano et al. concluded that the cumulative rectal D2cc threshold of even 75 Gy results in grade III or higher GI toxicity at a rate of 16.1%. Consequently, despite local control, the quality of life of cancer survivors may be poor [14].

EBRT dose escalation can be explored as an option because it aims at improving OS through the prevention of locoregional disease progression and elimination of possible foci for distant metastases. Over the past four decades, dose escalation with acceptable toxicity profiles has been achievable due to technological advancements in diagnostic imaging and radiotherapy techniques, leading to the improved delineation of the target volume. Dose escalation, in some settings such as carcinoma prostate, oesophageal cancer [15], and locally advanced pancreatic cancer [16], has been proven to be associated with OS benefit.

In this prospective study, we propose an alternative to the standard treatment practice, with EBRT dose escalation by using IG-IMRT and weekly concurrent cisplatin followed by reduced doses of BT in locally advanced cervical carcinoma [2018 revised International Federation of Gynaecology and Obstetrics staging (FIGO 2018) stage IIB-IIIC1] to overcome the challenge associated with BT applicator placement and treatment delivery. Our aim is to assess the response to EBRT dose escalation and the incidence of acute toxicity with IG-IMRT planning.

## Materials and methods

2.1. Study population

In total, 52 patients diagnosed with cervical cancer FIGO 2018 stage IIB-IIIC1 and treated from November 2019 to October 2021 at the Government Medical College and Cancer Hospital, Aurangabad, were invited to participate in the study.

2.2. Criteria for patient enrolment

**Figure 1:**
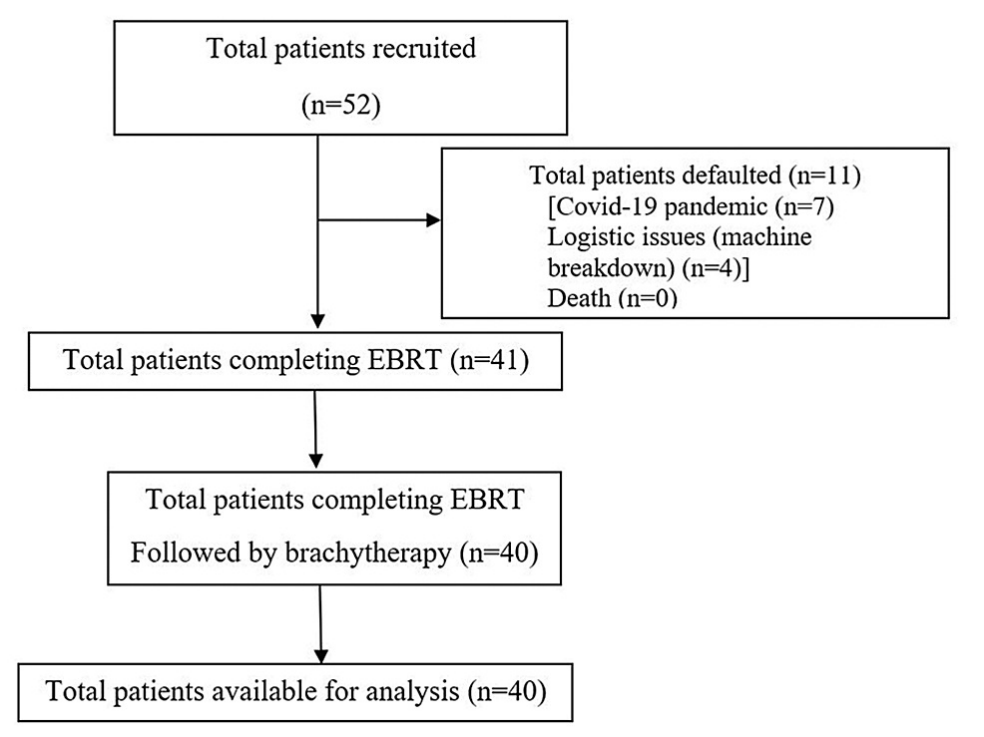
Population enrolment chart COVID-19: coronavirus disease 2019; EBRT: external beam radiation therapy

Eligibility criteria for the patients were as follows: women in the age group of 18-65 years; patients who received a biopsy-proven diagnosis of cervical carcinoma stage IIB-IIIC1 (squamous cell carcinoma or adenocarcinoma); patients with Eastern Cooperative Oncology Group (ECOG) [17] performance status 0-1; patients with baseline hemoglobin level >8 g/dL; those with normal leukocyte and platelet count; those with normal renal function; and those with no cardiac comorbidities. Patients with ECOG ≥2, severe anemia, and glomerular filtration rate <15 mL/min/m^2^ were excluded from the study. In total, 40 patients were included in the final analysis (Figure 1).

2.3. Study design and treatment

**Figure 2:**
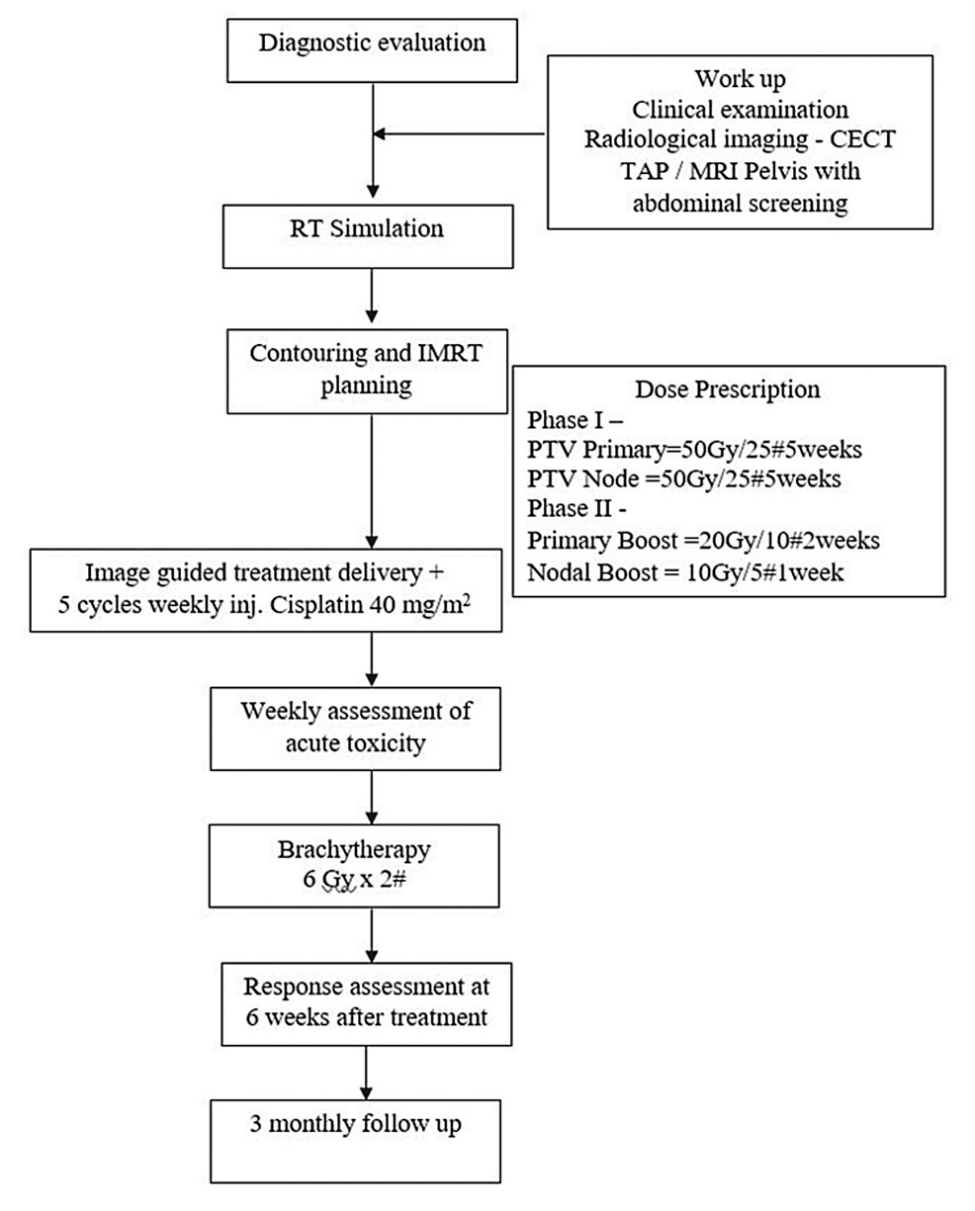
RT treatment protocol RT: radiation therapy; CECT: contrast-enhanced computed tomography; MRI: magnetic resonance imaging; PTV: planning target volume; IMRT: intensity-modulated radiation therapy

All eligible patients received diagnostic evaluation as per the standard guidelines after they provided their written informed consent. Patients were simulated in the head-first supine position after bladder protocol of 1 liter in 45 minutes and immobilization with a 4-clamp abdominopelvic thermoplastic mold (E1). Contrast-enhanced CT (CECT) images were acquired with 3-mm slice thickness from the level of the diaphragm to the mid-thigh (Figure 2).

Gross tumor volume (GTV) primary indicates the gross tumor volume; GTV node indicates pelvic LN of >1 cm; CTV primary includes GTV primary, uterine cervix, uterine corpus, parametrium, vagina, and ovaries; and CTV node indicates 7-mm isotropic margin to the GTV node and pelvic LNs. The planning target volume (PTV) was defined as CTV (primary + node) plus an isotropic margin of 3 mm. RT planning was performed using the IMRT technique with the Monte Carlo algorithm on the IMRT Elekta Synergy System, Monaco version 5.11.01 (Elekta, Stockholm, Sweden). The plan was evaluated and approved by a dedicated radiation oncologist.

The treatment setup was verified through onboard imaging in the form of cone-beam CT before the execution of the IMRT plan.

Phase I

Patients received 50 Gy in 25 fractions over five weeks at 2-Gy per fraction to PTV primary and draining LN with respectable normal tissue tolerance along with concurrent cisplatin administration to a dose of 40 mg/m^2^ once a week, with weekly monitoring of blood parameters.

Phase II

SEB to primary gross residual disease to a dose of 20 Gy in 10 fractions over two weeks. Sequential nodal boost to LNs >1 cm at EBRT conclusion to a total dose of 60 Gy in 30 fractions.

The patients were reviewed after every five fractions of RT to monitor acute treatment-related toxicities as per the RTOG criteria. One week after EBRT completion, the patients received intracavitary image-based BT with a total dose of 12 Gy in two fractions for two weeks. Treatment planning and delivery were executed using the Oncentra planning system microSelectron HDR v. 2, with 18 channels, version 4.5.2.

2.4. Patient assessment

The first follow-up was conducted six weeks after the completion of chemoradiation for response assessment with the complete pelvic examination and cross-sectional imaging such as CECT (abdomen and pelvis) or MRI (abdomen and pelvis). RECIST criteria version 1.1 was used for response assessment. The Common Terminology Criteria for Adverse Events (CTCAE) version 5 was used for grading hematologic toxicities, and RTOG criteria were used for assessing radiation-related toxicities. The patients were followed up every three months and examined clinically through imaging tests, as required.

The study protocol was duly approved by the institutional ethics committee, and the patients who refused to provide consent were excluded.

2.5. Statistical analysis

All data were prospectively maintained in the SPSS Statistics software for Windows, version 24.0 (IBM Corp., Armonk, NY). Quantitative data are presented as the arithmetic mean ±standard deviation (SD), whereas categorical data are presented as the frequency and percentage. The toxicity of dose-escalated IMRT was evaluated using Kaplan-Meier estimates. The Kaplan-Meier method is used to analyze “time to event data.” In the present study, the main outcome was the development of toxicities such as skin toxicity, GI toxicity, GU toxicity, and anemia after the initiation of dose-escalated IMRT at different time intervals over weeks. Kaplan-Meier graphs for toxicities such as skin, GI, and GU toxicities and anemia were plotted in the SPSS software.

## Results

The six-month follow-up data were available for 40 patients. One patient defaulted on the treatment protocol and was, therefore, excluded from the study. Patient- and treatment-related characteristics are listed in Table 1 and Table 2, respectively. The overall treatment time was calculated from the date of EBRT initiation to the date of the second BT.

**Table 1:** Patient characteristics FIGO: International Federation of Gynecology and Obstetrics

Characteristic	Value
Age at time of diagnosis, years	50 (45-56)
FIGO stage, n (%)	
IIB	7 (17.5%)
IIIA	2 (5%)
IIIB	22 (55%)
IIIC1	9 (22.5%)
Size of the primary tumor, n (%)	
<4 cm	0
>4 cm	40 (100%)
Lymph node status, n (%)	
<1 cm	8 (19.5%)
1-2 cm	8 (19.5%)
>2 cm	4 (9.7%)
Tumor histology, n (%)	
Squamous cell carcinoma	35 (87.5%)
Adenocarcinoma	3 (7.5%)
Adenosquamous	2 (5%)

**Table 2:** Treatment characteristics EBRT: external beam radiation therapy; BT: brachytherapy; SD: standard deviation

Characteristic	Value
Overall treatment time (EBRT + BT), mean ±SD	68.4 ±10.9
Number of cisplatin cycles taken, mean ±SD	4 ±6
Average dose of cisplatin received per patient	50.9 mg
Average dose received by 2-cc bladder during BT	5.5 Gy
Average dose received by 2-cc rectum during BT	7.7 Gy

The majority of the patients (35, 87.5%) were confirmed to have squamous cell carcinoma based on the histological analysis. Furthermore, 22 (55%) patients had FIGO 2018 stage IIIB. All patients had tumor diameters of >4 cm. The LN-positive status of size >1 cm at the baseline was noted in 12 patients. These 12 patients received a sequential nodal boost after the completion of 50 Gy in 25 fractions. We did not record any gap during the entire treatment period. All patients received at least four to six cycles of concurrent cisplatin to a dose of 40 mg/m^2^.

EQD2 of the dose administered to the primary tumor was comparable to the standardized RT dose fractionation schedule (Tables 3, 4). The true biological dose delivered through EBRT to the pelvis with the dose fractionation schedule of the current study was 103.2 Gy.

**Table 3:** BED and EQD2 in standard RT schedule BED: biologically effective dose; EQD2: equivalent total doses in 2-Gy fractions; RT: radiation therapy; PTV: planning target volume; SIB: simultaneous integrated boost; BT: brachytherapy

	Standard RT schedule	EQD2	BED
PTV primary	50 Gy/25#/5 weeks	50 Gy	60 Gy
PTV node (SIB)	55 Gy/25#/5 weeks	55.92 Gy	67.1 Gy
BT	7 Gy x 3#	29.75 Gy	35.7 Gy
Total (PTV primary + BT)		79.75 Gy	95.7 Gy

**Table 4:** BED and EQD2 in this study BED: biologically effective dose; EQD2: equivalent total doses in 2-Gy fractions; PTV: planning target volume; EBRT: external beam radiation therapy; BT: brachytherapy

	Current study	EQD2	BED
PTV primary + EBRT boost	70 Gy/35#/7 weeks	70 Gy	84 Gy
PTV node + sequential boost	60 Gy/30#/6 weeks	60 Gy	72 Gy
BT	6 Gy x 2#	16 Gy	19.2 Gy
Total (PTV primary + BT)		86 Gy	103.2 Gy

Dosimetric characteristics are presented in Table 5 and Figure 4. The mean ±SD doses received by 15% and 50% volume of the rectum were 63.26 ±4.29 and 51.42 ±6.30 Gy, respectively. The mean ±SD doses received by 15% and 50% volume of the urinary bladder were 60.94 ±4.96 and 46.47 ±6.51 Gy, respectively. The doses received by 195 cc and 150 cc of the bowel bag were 35.26 ±6.83 and 35.26 ±6.83 Gy, respectively. Both the femoral heads received the maximum dose of 33.7 Gy.

The average doses received by 2-cc volume of the urinary bladder and 2-cc volume of the rectum with one fraction of BT were 5.5 and 7.7 Gy, respectively (Figure 5).

**Table 5:** Dose constraints as per RTOG 0415 study RTOG: the Radiation Therapy Oncology Group; PTV: planning target volume; CTV: clinical target volume; SD: standard deviation

Volume	Dose as per RTOG 0415	Current study dose (Gy), mean ±SD
PTV (max)		74.15 ±0.83
PTV (min)	(>98% should receive 70 Gy)	68.73 ±0.93
CTV (min)	(>100% should receive 70 Gy)	64.52 ±3.02
Bladder V <15%	80 Gy	60.94 ±4.96
Bladder V <25%	75 Gy	56.68 ±5.43
Bladder V <35%	70 Gy	52.73 ±5.72
Bladder V <50%	40 Gy	46.47 ±6.51
Rectum V <15%	75 Gy	63.26 ±4.29
Rectum V <25%	70 Gy	59.63 ±5.20
Rectum V <35%	65 Gy	56.15 ±5.76
Rectum V <50%	60 Gy	51.42 ±6.30
Femur Dmax	-	34.6 ±6.3
Bowel Bag 150 cc		38.05 ±6.97
Bowel Bag 195 cc		35.26 ±6.83

**Figure 3:**
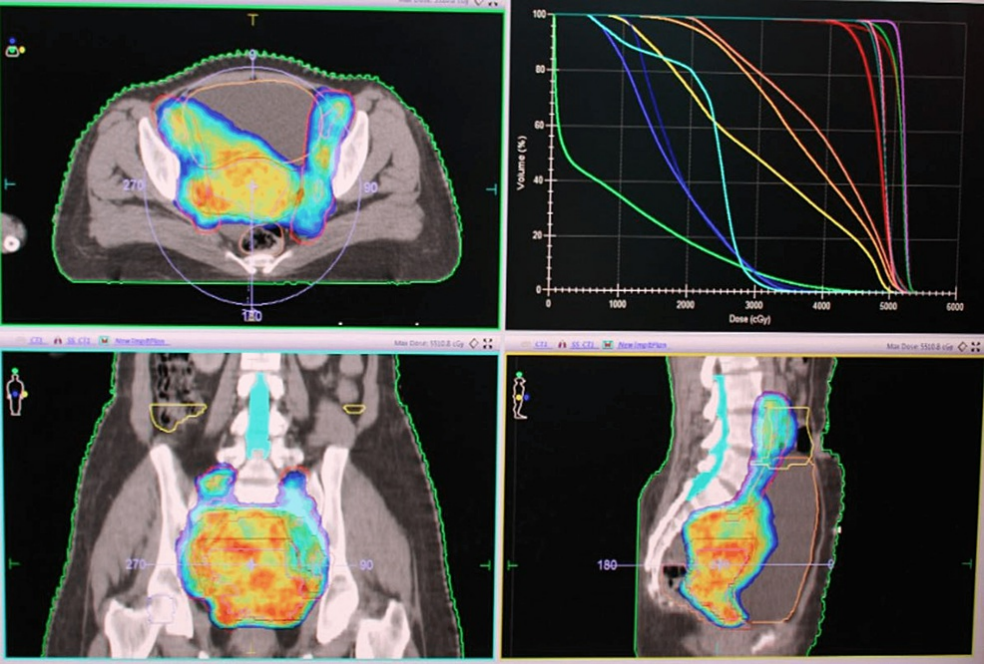
Planning system Monaco version 5.11.01 - 95% isodose distribution

**Figure 4:**
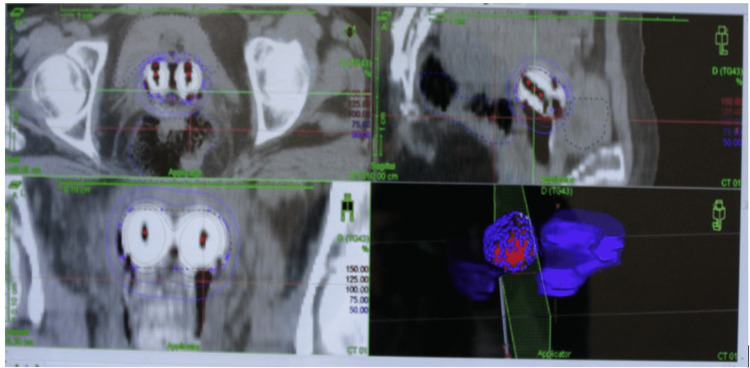
Brachytherapy dose distribution

Figure 3 illustrates the planning system Monaco version 5.11.01 - 95% isodose distribution. Figure 4 depicts BT dose distribution.

3.1. Treatment-related toxicity

**Figure 5:**
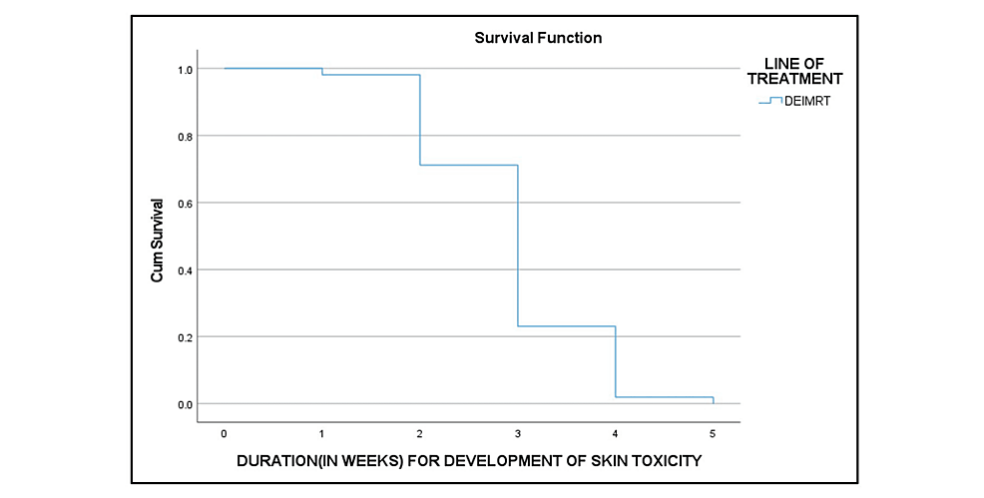
Kaplan-Meier curve of the duration for the development of acute skin toxicity

**Figure 6:**
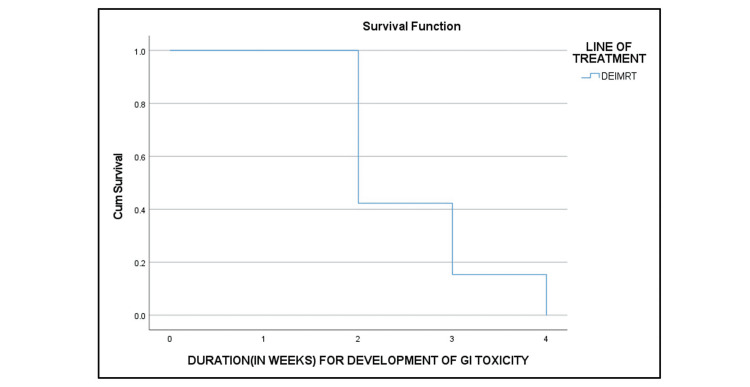
Kaplan-Meier curve of the duration for the development of acute GI toxicity GI: gastrointestinal

**Figure 7:**
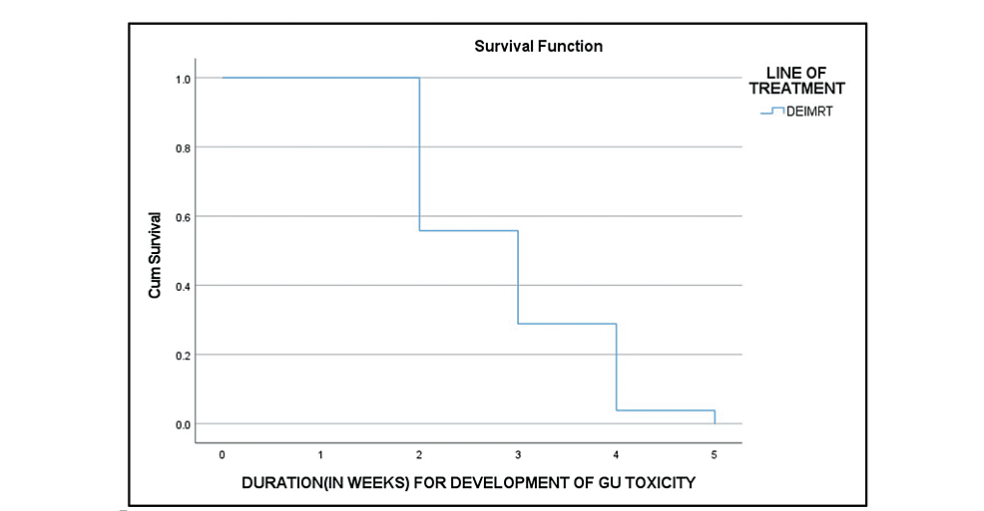
Kaplan-Meier curve of the duration for the development of acute GU toxicity GU: genitourinary

RTOG grade III dermatitis (Table 6 and Figure 5), grade III acute GI toxicity (Figure 6), and grade III acute GU toxicity (Figure 7) were observed in three (7.5%), six (15%), and three (7.5%) patients, respectively. None of the patients had grade IV toxicity.

**Table 6:** RTOG acute toxicity at CTRT completion RTOG: the Radiation Therapy Oncology Group

Acute toxicity	At CTRT completion
Dermatitis	Grade I – 18 (45%)
	Grade II – 19 (47.5%)
	Grade III – 3 (7.5%)
Gastrointestinal toxicity	Grade I – 9 (22%)
	Grade II – 25 (60%)
	Grade III – 6 (17.5%)
Genitourinary toxicity	Grade I – 21 (52.5%)
	Grade II – 16 (40%)
	Grade III – 3 (7.5%)

CTCAE grade I anemia was observed in all patients. White blood cell (WBC) counts, platelet counts, and serum creatinine levels were within normal limits during CTRT treatment. Anemia resolved within six weeks of RT completion (Table 7).

**Table 7:** Blood investigation profile SD: standard deviation

Acute toxicity	At CTRT completion
Hemoglobin levels, g/dL, mean +SD	9.84 ±1.39
Total WBC count, mean ±SD	5.86 ±1.57
Total platelet count, mean ±SD	2.14 ±0.99
Serum creatinine, mg/dl, mean ±SD	1.1 ±0.9

**Figure 8:**
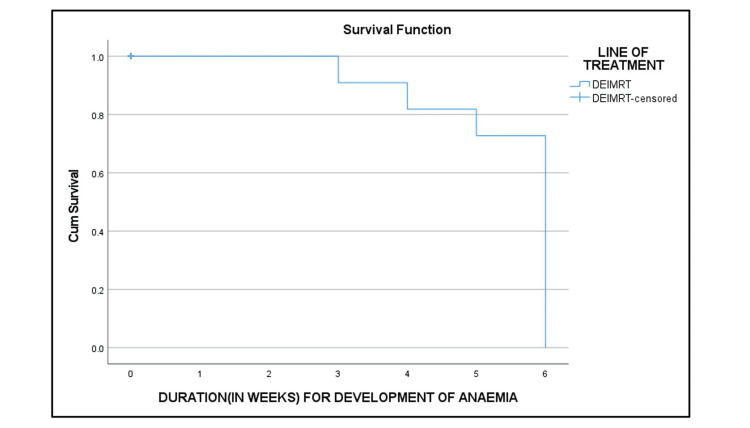
Kaplan-Meier curve of the duration for the development of anemia

Figure 8 shows the Kaplan-Meier curve of the duration for the development of anemia.

3.2. Response rate

Assessment at six weeks after treatment completion demonstrated complete response in 26 (65%) patients and partial response in 14 (35%) patients. At three and six months, seven (17.5%) and three (7.5%) patients, respectively, had residual lesions (Table 8).

**Table 8:** Clinicoradiologic response assessment

Cancer stage response assessment	At 6 weeks, n (%)	At 3 months, n (%)	At 6 months, n (%)
Partial response	15 (36.6%)	7 (17.5%)	3 (7.5%)
Complete response	26 (63.4%)	33 (82.5%)	37 (92.5%)
Stable disease	-	-	-
Progressive disease	-	-	-

## Discussion

IG-IMRT is a highly conformal technique that demonstrates an improved therapeutic ratio, and therefore, it offers the possibility of sparing adjacent normal tissues without compromising the target volume coverage [18]. The treatment of gynecologic malignancies with IG-IMRT has been on the rise in the past decade despite the dearth of data on late toxicities and disease control outcomes. Moreover, little is known regarding EBRT dose intensification with de-escalated BT. The Meta-Analysis Group of the Medical Research Council Clinical Trials Unit analyzed 13 trials comparing concurrent CTRT with chemotherapy alone in patients diagnosed with FIGO stage IB-IVA cancer receiving an RT dose ranging from 40 to 45 Gy to the whole pelvis with EBRT boost to primary up to 61.2 Gy followed by BT boost ranging from 18 to 50 Gy [19]. Remarkable improvements of 8% and 9%, respectively, were observed in terms of five-year disease-free survival (DFS) and locoregional control, with CTRT regimen at the cost of a higher rate of GI toxicity because conventional techniques were used in most of the trials. Traditionally, pelvic EBRT doses were limited to 45-50 Gy with conventional techniques primarily due to the small bowel tolerance being a limiting factor. Dosimetric studies from several retrospective cohorts have shown IMRT to be superior to the conventional technique in terms of GI and GU toxicity profiles. Mundt et al. reported a significantly lower grade of GI toxicity (grade II) with the IMRT technique compared to the conventional technique (3% vs. 20%) [20]. The use of dose-escalated EBRT with the IMRT technique followed by de-escalated BT by Nikola et al. showed a >25% incidence of grade III or higher GI and GU toxicities with conventional CTRT [21]. In our study, we observed similar acceptable rates of acute grade III GI toxicity in five (12.5%) patients and acute grade III GU toxicity in three (7.5%) patients at the sixth week of CTRT treatment.

Nikola et al. planned EBRT for primary tumors with a dose of 50.4 Gy, and for tumors >4 cm in diameter, an additional EBRT boost of 9 Gy was administered. The total HDR-BT dose ranged from 15 to 18 Gy. At a median follow-up of 35.5 months, despite dose escalations, nine (23.1%) patients developed locoregional recurrence, with seven (77.7%) of them being in-field recurrence. In our study, all patients had tumor diameters of >4 cm, and we delivered SEB to primary tumors at a dose of 20 Gy compared with 10 Gy planned by Nikola et al., because radiobiologically, primary tumors generally have a necrotic component with a hypoxic radioresistant focus that requires higher lethal doses for tumor cell killing. The total dose received by the LN was 60 Gy in our study, which followed the NCCN 2022 recommendations of an LN boost of 56-63 Gy.

Dosimetric data from the randomized control dose-escalation trial in patients with prostate cancer performed at MD Anderson Cancer Center revealed that the risk of rectal bleeding decreased from 46% to 6% when <25% of the rectum received a 70-Gy dose [22]. This constraint has become the standard for subsequent studies such as the RTOG 9406 3DCRT study and RTOG 0415 phase III randomized study of hypo-fractionated 3DCRT/IMRT versus conventionally fractionated 3DCRT/IMRT treatment in patients with favorable-risk prostate cancer. We adopted the same dose-constraint protocol and found that <35% of the rectum received a dose of 56.15 ±5.76 Gy. Furthermore, <60 Gy was received by >50% of the rectal volume. This was in concordance with the RTOG 0415 dose constraints.

In this study, we hypothesized that an escalated dose for primary and draining pelvic LNs is essential to treat the macroscopic and subclinical primary tumor and lymph nodal disease to improve DFS and locoregional control. Furthermore, with an escalated EBRT dose, the bulk of the primary tumor is reduced, improving the chances of BT applicator placement with ideal geometry. Another reason for delivering dose-escalated EBRT to the pelvis is the fact that the cervix and uterus can tolerate >200 Gy. With this dose, the rate of necrosis is <1%. The BED of 103.3 Gy in our study is lower than the reported threshold doses of 150 Gy for vesicovaginal fistula [23].

Because the present study was conducted at a single center and involved a small sample size and noncomparative design, we recommend a randomized control study with a larger target population group to validate the CTRT treatment protocol proposed in our study. Another limitation of our study is the disparity in the cross-sectional imaging used for staging in the target population. In the wake of the coronavirus disease pandemic and our institute being a government cancer center, positron emission tomography/CT was not performed for the baseline evaluation of LNs and disease extent during the study period. At present, our study is in the initial phases of follow-up, and hence, we do not have data regarding late toxicity, five-year OS, DFS, and LRC rates. Nevertheless, we believe that this first-of-its-kind study in the Indian population with a favorable toxicity profile holds promise for the management of cervical carcinoma in the future.

## Conclusions

Our findings revealed that patients with cervical cancer treated with dose-escalated IMRT have satisfactory outcomes with reasonably low levels of treatment-related acute GI and GU toxicities. The present study indicates that the application of a high dose of external radiation to the pelvis by using the IMRT technique with image-guided delivery could be an acceptable alternative to the standard-dose management schedule.

## References

[1] Sung H, Ferlay J, Siegel RL, Laversanne M, Soerjomataram I, Jemal A, Bray F (2021). Global Cancer Statistics 2020: GLOBOCAN estimates of incidence and mortality worldwide for 36 cancers in 185 countries. CA Cancer J Clin.

[2] (2022). Globocan 2020 India cancer statistics. https://gco.iarc.fr/today/data/factsheets/populations/900-world-fact-sheets.pdf.

[3] Mazeron R, Petit C, Rivin E (2016). Which is the optimal radiotherapy pelvic dose in locally advanced cervical cancer in the perspective of reaching magnetic resonance image-guided adaptive brachytherapy planning aims?. Clin Oncol (R Coll Radiol).

[4] Tanderup K, Eifel PJ, Yashar CM, Pötter R, Grigsby PW (2014). Curative radiation therapy for locally advanced cervical cancer: brachytherapy is NOT optional. Int J Radiat Oncol Biol Phys.

[5] Kirwan JM, Symonds P, Green JA, Tierney J, Collingwood M, Williams CJ (2003). A systematic review of acute and late toxicity of concomitant chemoradiation for cervical cancer. Radiother Oncol.

[6] Gerstner N, Wachter S, Knocke TH, Fellner C, Wambersie A, Pötter R (1999). The benefit of Beam's eye view based 3D treatment planning for cervical cancer. Radiother Oncol.

[7] Eifel PJ, Thoms WW Jr, Smith TL, Morris M, Oswald MJ (1994). The relationship between brachytherapy dose and outcome in patients with bulky endocervical tumors treated with radiation alone. Int J Radiat Oncol Biol Phys.

[8] Endo D, Todo Y, Okamoto K, Minobe S, Kato H, Nishiyama N (2015). Prognostic factors for patients with cervical cancer treated with concurrent chemoradiotherapy: a retrospective analysis in a Japanese cohort. J Gynecol Oncol.

[9] (2022). NCCN: Cervical Cancer. https://www.nccn.org/professionals/physician_gls/pdf/cervical_blocks.pdf.

[10] Dutta S, Nguyen NP, Vock J (2015). Image-guided radiotherapy and brachytherapy for cervical cancer. Front Oncol.

[11] Bonin SR, Lanciano RM, Corn BW, Hogan WM, Hartz WH, Hanks GE (1996). Bony landmarks are not an adequate substitute for lymphangiography in defining pelvic lymph node location for the treatment of cervical cancer with radiotherapy. Int J Radiat Oncol Biol Phys.

[12] Liberman D, Mehus B, Elliott SP (2014). Urinary adverse effects of pelvic radiotherapy. Transl Androl Urol.

[13] Viswanathan AN, Beriwal S, De Los Santos JF (2012). American Brachytherapy Society consensus guidelines for locally advanced carcinoma of the cervix. Part II: high-dose-rate brachytherapy. Brachytherapy.

[14] Romano KD, Hill C, Trifiletti DM (2018). High dose-rate tandem and ovoid brachytherapy in cervical cancer: dosimetric predictors of adverse events. Radiat Oncol.

[15] Ghosh Laskar S, Mummudi N, Kumar S (2021). Definitive radiation therapy with dose escalation is beneficial for patients with squamous cell cancer of the esophagus (Epub ahead of print). J Can Res Ther.

[16] Koay EJ, Hanania AN, Hall WA (2020). Dose-escalated radiation therapy for pancreatic cancer: a simultaneous integrated boost approach. Pract Radiat Oncol.

[17] Oken MM, Creech RH, Tormey DC, Horton J, Davis TE, McFadden ET, Carbone PP (1982). Toxicity and response criteria of the Eastern Cooperative Oncology Group. Am J Clin Oncol.

[18] Hong TS, Ritter MA, Tomé WA, Harari PM (2005). Intensity-modulated radiation therapy: emerging cancer treatment technology. Br J Cancer.

[19] Chemoradiotherapy for Cervical Cancer Meta-Analysis Collaboration (2008). Reducing uncertainties about the effects of chemoradiotherapy for cervical cancer: a systematic review and meta-analysis of individual patient data from 18 randomized trials. J Clin Oncol.

[20] Mundt AJ, Lujan AE, Rotmensch J, Waggoner SE, Yamada SD, Fleming G, Roeske JC (2002). Intensity-modulated whole pelvic radiotherapy in women with gynecologic malignancies. Int J Radiat Oncol Biol Phys.

[21] Cihoric N, Tsikkinis A, Tapia C, Aebersold DM, Zlobec I, Lössl K (2015). Dose escalated intensity modulated radiotherapy in the treatment of cervical cancer. Radiat Oncol.

[22] Buckey CR, Swanson GP, Stathakis S, Papanikolaou N (2010). Optimizing prostate intensity-modulated radiation therapy (IMRT): do stricter constraints produce better dosimetric results?. Eur J Clin Med Oncol.

[23] Shrivastava SK, Mahantshetty U, Narayan K (2015). Principles of radiation therapy in low-resource and well-developed settings, with particular reference to cervical cancer. Int J Gynaecol Obstet.

